# Supplementary material for: Discovery of a drug candidate for GLIS3-associated diabetes
Source: Nat Commun. 2018 Jul 11;9:2681. doi: 10.1038/s41467-018-04918-x (PMC6041295; doi:10.1038/s41467-018-04918-x)
Supplement: Supplementary file 1 — Supplementary Information [file 41467_2018_4918_MOESM1_ESM.pdf]

SUPPLEMENTARY INFORMATION

Title: Discovery of a Drug Candidate for GLIS3-Associated Diabetes

Amin et al.

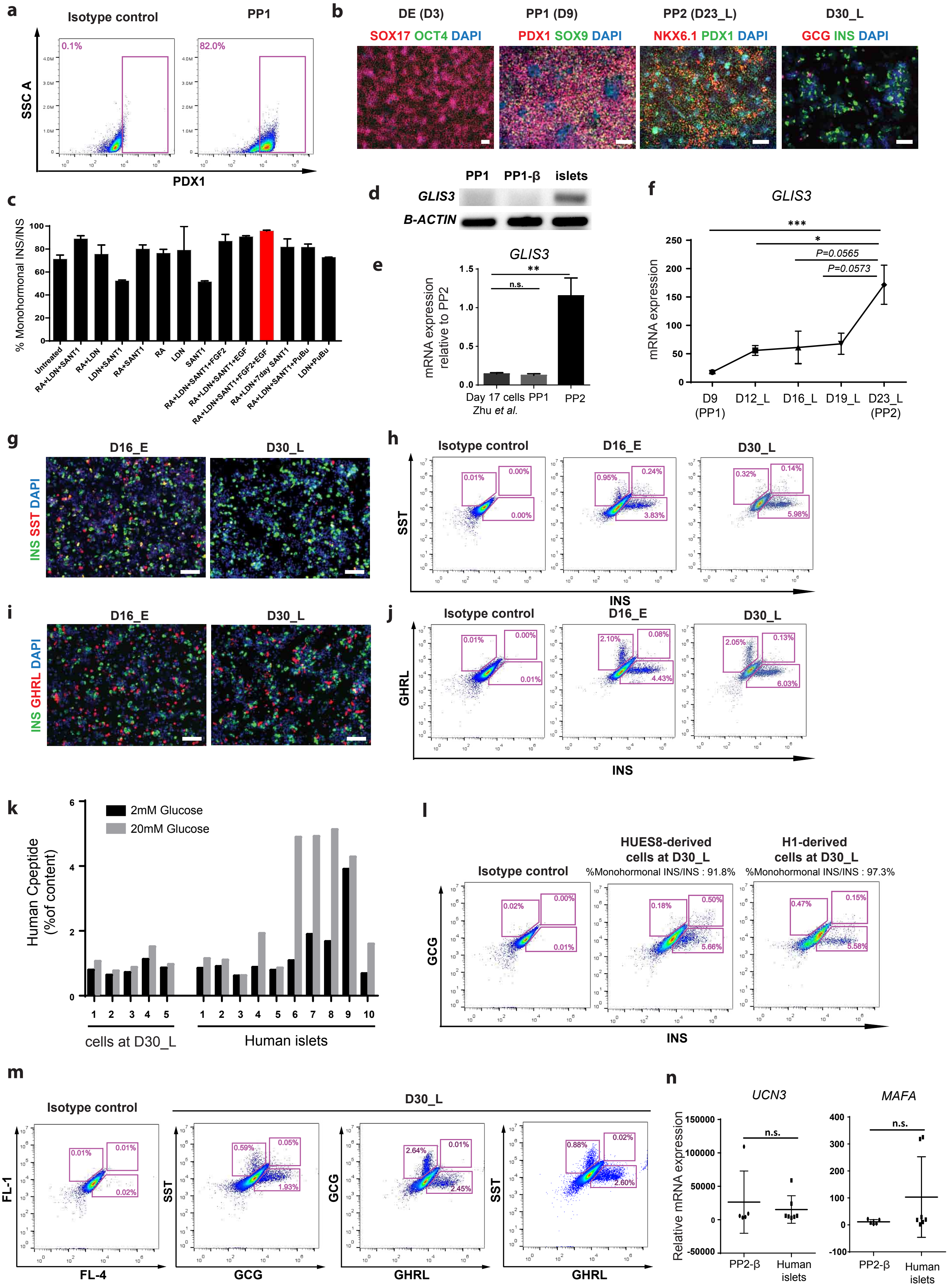
a
Isotype control
PP1
0.1%
82.0%
SSC A
PDX1
b
DE (D3)
PP1 (D9)
PP2 (D23_L)
D30_L
SOX17 OCT4 DAPI
PDX1 SOX9 DAPI
NKX6.1 PDX1 DAPI
GCG INS DAPI
c
% Monohormonal INS/INS
Untreated
RA+LDN+SANT1
RA+LDN
LDN+SANT1
RA+SANT1
RA
LDN
SANT1
RA+LDN+SANT1+FGF2
RA+LDN+SANT1+EGF
RA+LDN+SANT1+FGF2+EGF
RA+LDN+7day SANT1
RA+LDN+SANT1+PuBu
LDN+PuBu
d
PP1
PP1-β
islets
GLIS3
B-ACTIN
e
GLIS3
mRNA expression relative to PP2
n.s.
**
Day 17 cells Zhu et al.
PP1
PP2
f
GLIS3
***
*
P=0.0565
P=0.0573
mRNA expression
D9 (PP1)
D12_L
D16_L
D19_L
D23_L (PP2)
g
D16_E
D30_L
INS SST DAPI
h
Isotype control
D16_E
D30_L
0.01%
0.00%
0.00%
0.95%
0.24%
3.83%
0.32%
0.14%
5.98%
SST
INS
i
D16_E
D30_L
INS GHRL DAPI
j
Isotype control
D16_E
D30_L
0.01%
0.00%
0.01%
2.10%
0.08%
4.43%
2.05%
0.13%
6.03%
GHRL
INS
k
2mM Glucose
20mM Glucose
Human Cpeptide (%of content)
cells at D30_L
Human islets
l
Isotype control
HUES8-derived cells at D30_L
%Monohormonal INS/INS : 91.8%
H1-derived cells at D30_L
%Monohormonal INS/INS : 97.3%
0.02%
0.00%
0.01%
0.18%
0.50%
5.66%
0.47%
0.15%
5.58%
GCG
INS
m
Isotype control
D30_L
0.01%
0.01%
0.02%
0.59%
0.05%
1.93%
2.64%
0.01%
2.45%
0.88%
0.02%
2.60%
FL-1
FL-4
SST
GCG
GCG
GHRL
SST
GHRL
n
UCN3
MAFA
n.s.
n.s.
Relative mRNA expression
PP2-β
Human islets

Supplementary Figure 1. A pilot screen to establish the protocol to generate late stage pancreatic progenitors that give rise to mono-hormonal PP2-β cells.

(a) Intracellular flow cytometry for PDX1 expression in PP1 cells. **(b)** Representative images for DE, PP1, PP2 cells and cells at D30_L. Scale bar=100 µm. **(c)** The percentage of mono-hormonal ($INS^{+}/GCG^{-}SST^{-}$) cells in INS^{+} cells derived in different culture media conditions (n=2). **(d)** RT-PCR analysis of *GLIS3* expression in PP1, PP1-β and human islets. **(e)** qRT-PCR analysis of GLIS3 expression in day 17 (PH-β) cells from Zhu *et al*, PP1 and PP2 cells. (PH-β n=4, PP1 and PP2 n=6). **(f)** Time-course qRT-PCR analysis of GLIS3 expression during transition from PP1 to PP2 stages of differentiation. (Data are normalized to ES; PP1, PP2 n=6, day 12, 16, 19 n=4). **(g)** Immunocytochemistry analysis of INS and SST expression at D16_E and D30_L. Scale bar=100 µm. **(h)** Intracellular flow cytometry analysis of INS and SST expression at D16_E and D30_L. **(i)** Immunocytochemistry analysis of INS and GHRL expression at D16_E and D30_L. Scale bar=100 µm. **(j)** Intracellular flow cytometry analysis for INS and GHRL expression at D16_E and D30_L. **(k)** C-peptide secretion (% of total c-peptide content) in response to 2 mM (low) and 20 mM (high) D-glucose conditions. Data are shown for individual batches of cells at D30_L and human islets (PP2-β n=5, Human islets n=10). **(l)** Intracellular flow cytometry analysis of INS and GCG expression in cells at D30_L derived from HUES8 and H1 hESCs. **(m)** Intracellular flow cytometry analysis for co-expression of different endocrine hormones (GCG, SST and GHRL) at D30_L. **(n)** qRT-PCR analysis of *UCN3* and *MAFA* in PP2-β cells and human primary islets (PP2-β n=5, Human islets n=7). *P* values by multiple unpaired student t-test were *$P<0.05$, **$P<0.01$, ***$P<0.001$, ****$P<0.0001$, n.s. not significant. The center value is “mean”. Error bar is SEM.

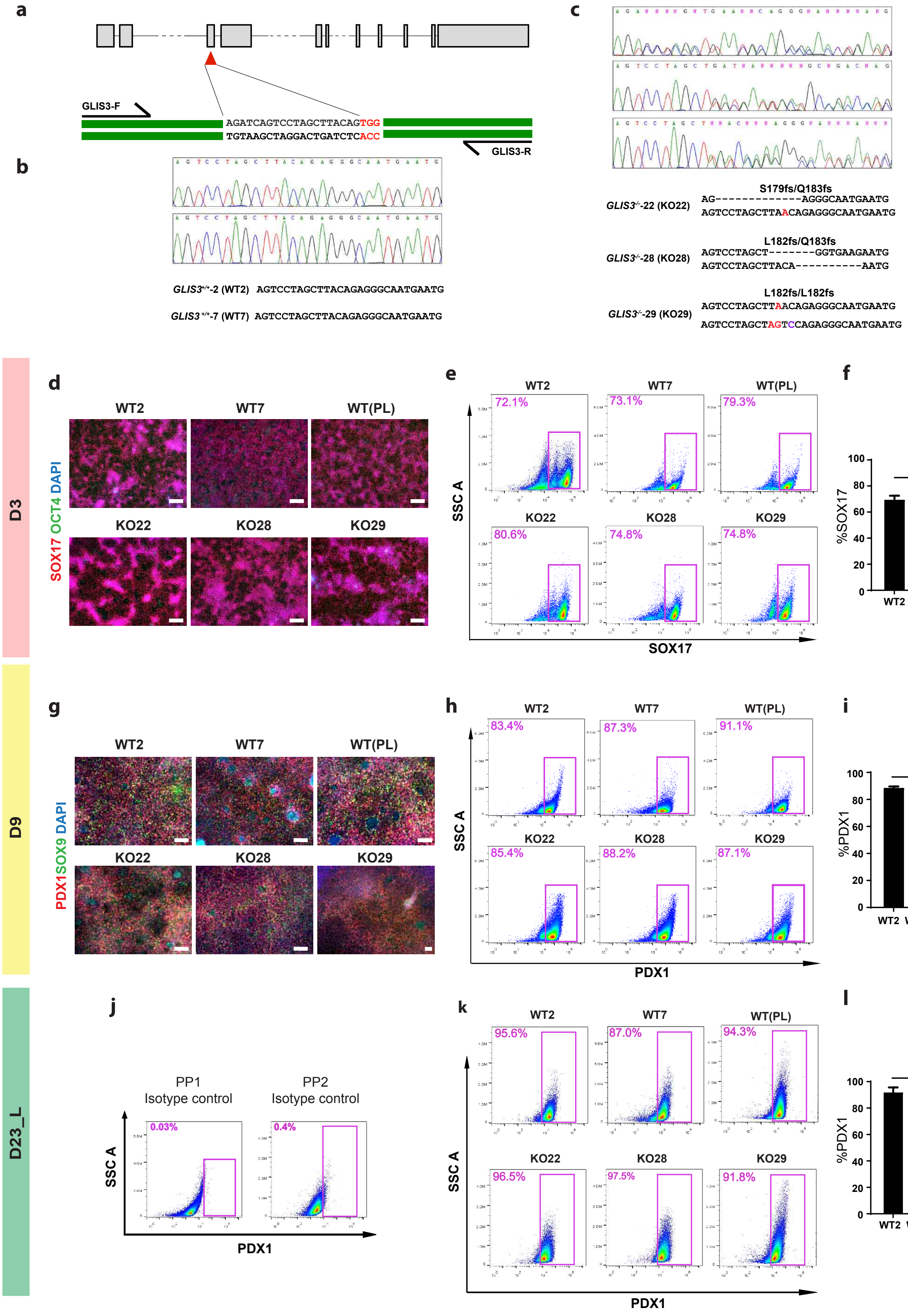
a
GLIS3-F
AGATCAGTCCTAGCTTACAGTGG
TGTAAGCTAGGACTGATCTCACC
GLIS3-R
b
GLIS3+/+-2 (WT2) AGTCCTAGCTTACAGAGGGCAATGAATG
GLIS3+/+-7 (WT7) AGTCCTAGCTTACAGAGGGCAATGAATG
c
S179fs/Q183fs
GLIS3-/--22 (KO22)
AG-------------AGGGCAATGAATG
AGTCCTAGCTTAACAGAGGGCAATGAATG
L182fs/Q183fs
GLIS3-/--28 (KO28)
AGTCCTAGCT-------GGTGAAGAATG
AGTCCTAGCTTACA----------AATG
L182fs/L182fs
GLIS3-/--29 (KO29)
AGTCCTAGCTTAACAGAGGGCAATGAATG
AGTCCTAGCTAGTCCAGAGGGCAATGAATG
D3
d
SOX17 OCT4 DAPI
WT2
WT7
WT(PL)
KO22
KO28
KO29
e
SSC A
SOX17
72.1%
73.1%
79.3%
80.6%
74.8%
74.8%
f
DE
WT
KO
n.s.
%SOX17
D9
g
PDX1SOX9 DAPI
h
SSC A
PDX1
83.4%
87.3%
91.1%
85.4%
88.2%
87.1%
i
PP1
%PDX1
D23_L
j
PP1
Isotype control
PP2
Isotype control
0.03%
0.4%
k
95.6%
87.0%
94.3%
96.5%
97.5%
91.8%
l
PP2

Supplementary Figure 2. Generation and characterization of *GLIS3*$^{-/-}$ hESC lines.

(a) Schematic representation of the targeting strategy to induce frameshift mutations in exon 3 of the *GLIS3* gene. **(b)** Sequencing results of two distinct WT hESC clones. **(c)** Sequencing results of three distinct *GLIS3*$^{-/-}$ hESC clones. **(d)** Immunocytochemistry analysis of WT and *GLIS3*$^{-/-}$ DE cells. Scale bar=200 µm. **(e, f)** Intracellular flow cytometry analysis (e), and quantification (f) of WT and *GLIS3*$^{-/-}$ DE cells at D3. (WT2 and KO29 n=4, all other lines n=3) **(g)** Immunocytochemistry analysis of WT and *GLIS3*$^{-/-}$ cell at D9. Scale bar=100 µm. **(h, i)** Intracellular flow cytometry analysis (h), and quantification (i) of WT and *GLIS3*$^{-/-}$ cells at D9. (n=3 for WT7, n=1 for WT (PL), n=4 for all other lines). **(j)** Isotype control plots for PDX1 staining at D9 and D23_L. **(k, l)** Intracellular flow cytometry analysis (k) and the quantification (l) of WT and *GLIS3*$^{-/-}$ lines at D23_L (WT lines n=2, *GLIS3*$^{-/-}$ lines n=3). *P* values by multiple unpaired student t-test were *$P<0.05$, **$P<0.01$, ***$P<0.001$, ****$P<0.0001$, n.s. not significant. The center value is "mean". Error bar is SEM.

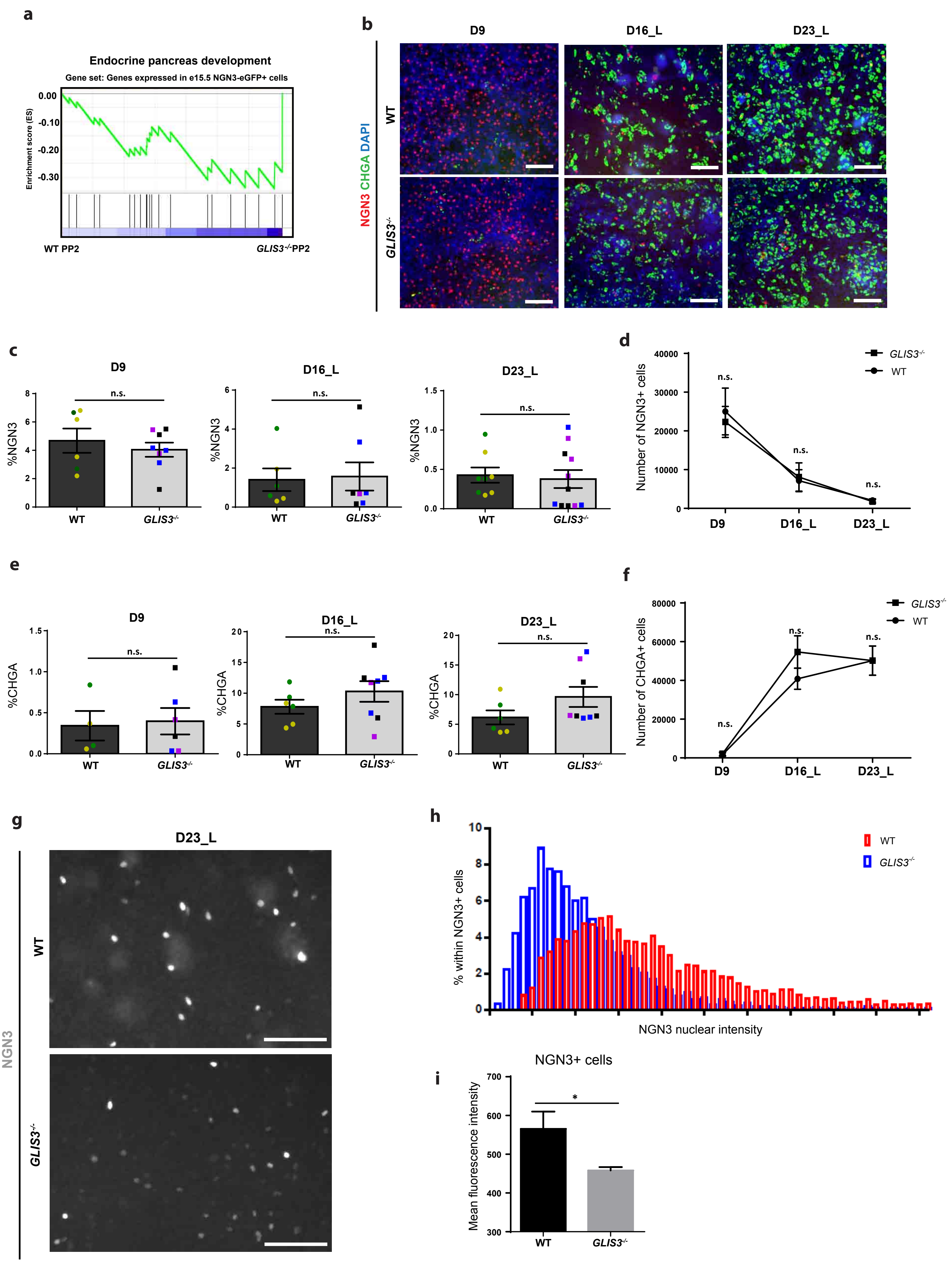

a
Endocrine pancreas development
Gene set: Genes expressed in e15.5 NGN3-eGFP+ cells
Enrichment score (ES)
WT PP2
GLIS3-/-PP2
b
D9
D16_L
D23_L
WT
GLIS3-/-
NGN3 CHGA DAPI
c
%NGN3
n.s.
d
Number of NGN3+ cells
e
%CHGA
f
Number of CHGA+ cells
g
NGN3
h
% within NGN3+ cells
NGN3 nuclear intensity
i
NGN3+ cells
Mean fluorescence intensity
*

Supplementary Figure 3. Quantification and characterization of the endocrine progenitors and their derivatives.

(a) GSEA analysis showing the decrease of endocrine pancreas-related genes in *GLIS3*$^{-/-}$ cells at D23_L (n=3). **(b)** Representative immunostaining images for NGN3 and CHGA staining on D9, D16_L and D23_L of the differentiation to derive late stage pancreatic progenitors. Scale bar= 100 µm. **(c, d)** Immunostaining quantification of the percentage (c) and number (d) of NGN3^{+} cells in WT and *GLIS3*$^{-/-}$ cells D9, D16_L and D23_L of differentiation. (WT, n=6 for D9 and D16_L, n=9 for D23_L, *GLIS3*$^{-/-}$, n=8 for D9 and D16_L, n=11 for D23_L). **(e, f)** Immunostaining quantification of the percentage (e) and number (f) of CHGA^{+} cells in WT and *GLIS3*$^{-/-}$ on D9, D16_L and D23_L of differentiation. (WT, n=4 for D9 and D16_L, n=6 for D23_L, *GLIS3*$^{-/-}$, n=6 for D9 and D16_L, n=8 for D23_L). (**g**) Representative images of NGN3 immunostaining in WT and *GLIS3*$^{-/-}$ cells at D23_L. Scale bar= 100 µm. (**h**) Distribution of NGN3 fluorescence intensity among NGN3^{+} WT and *GLIS3*$^{-/-}$ cells at D23_L. (**i**) Mean fluorescence intensity values for NGN3 immunostaining in WT and *GLIS3*$^{-/-}$ cells at D23_L (WT n=4, *GLIS3*$^{-/-}$ n=5).

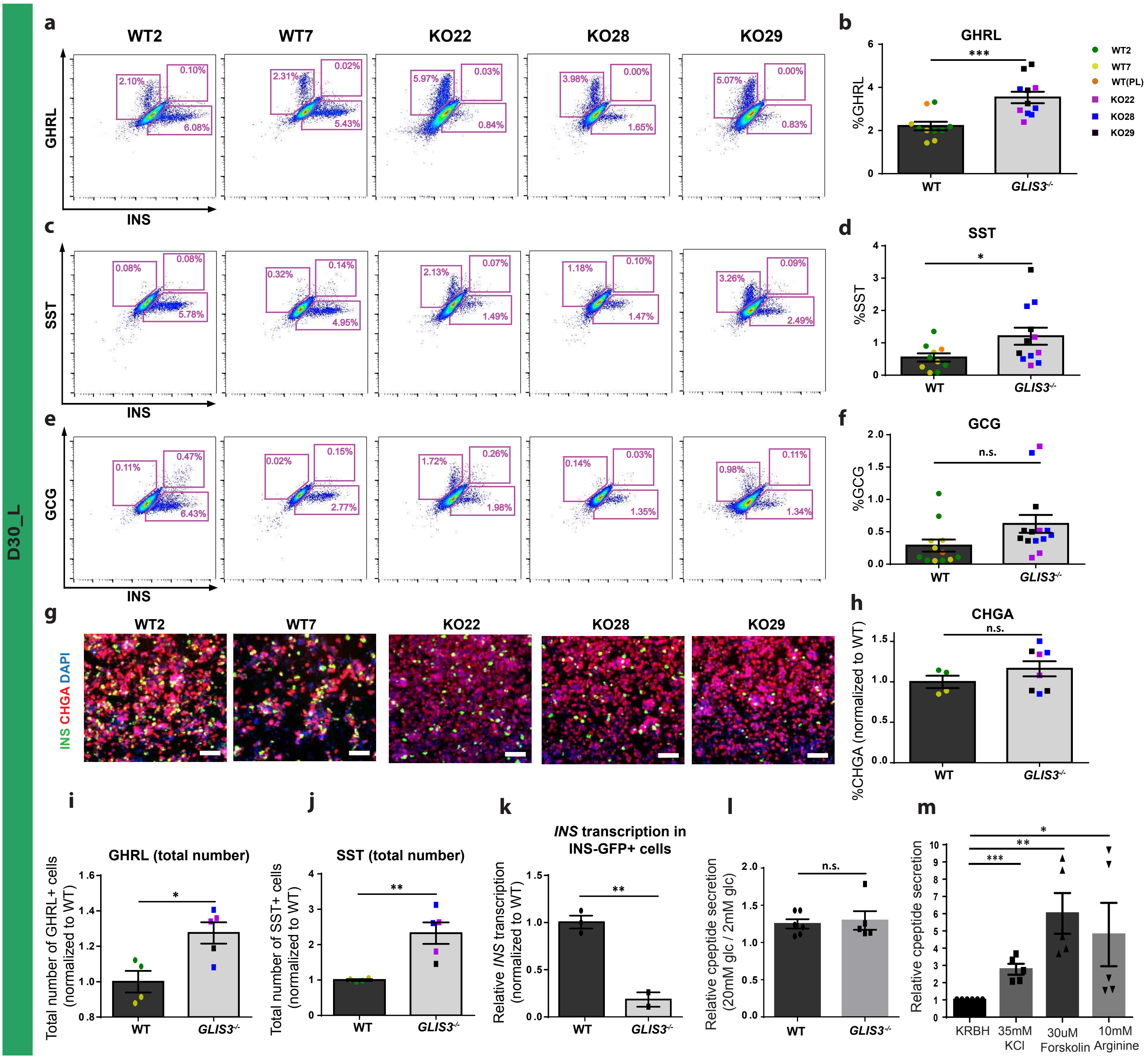
D30_L
a
WT2
WT7
KO22
KO28
KO29
GHRL
INS
b
GHRL
%GHRL
WT
GLIS3-/-
WT2
WT7
WT(PL)
KO22
KO28
KO29
c
SST
INS
d
SST
%SST
e
GCG
INS
f
GCG
%GCG
n.s.
g
INS CHGA DAPI
h
CHGA
%CHGA (normalized to WT)
i
GHRL (total number)
Total number of GHRL+ cells (normalized to WT)
j
SST (total number)
Total number of SST+ cells (normalized to WT)
k
INS transcription in INS-GFP+ cells
Relative INS transcription (normalized to WT)
l
Relative cpeptide secretion (20mM glc / 2mM glc)
m
Relative cpeptide secretion
KRBH
35mM KCl
30uM Forskolin
10mM Arginine

Supplementary Figure 4. Characterization of *GLIS3*$^{-/-}$ endocrine cells at D30_L.

(a) Intracellular flow cytometry analysis of INS and GHRL expression in WT and *GLIS3*$^{-/-}$ cells at D30_L. **(b)** Quantification of the percentage of GHRL^{+} cells in WT and *GLIS3*$^{-/-}$ cells at D30_L (WT n=10, *GLIS3*$^{-/-}$ n=12). **(c)** Intracellular flow cytometry analysis of INS and SST expression in WT and *GLIS3*$^{-/-}$ cells at D30_L. **(d)** Quantification of the percentage of SST^{+} cells in WT and *GLIS3*$^{-/-}$ cells at D30_L. (WT n=10, *GLIS3*$^{-/-}$ n=12). **(e)** Intracellular flow cytometry analysis of INS and GCG expression in WT and *GLIS3*$^{-/-}$ cells at D30_L. **(f)** Quantification of the percentage of GCG^{+} cells in WT and *GLIS3*$^{-/-}$ cells at D30_L. (WT n=12, *GLIS3*$^{-/-}$ n=14). **(g)** Immunocytochemistry analysis of INS and CHGA expression in WT and *GLIS3*$^{-/-}$ cells at D30_L. Scale bar=100 μm. **(h)** Quantification of the percentage of CHGA^{+} cells in WT and *GLIS3*$^{-/-}$ cells at D30_L. The values are normalized to the WT mean. **(i, j)** Relative number of GHRL^{+} (i) and SST^{+} cells in WT and *GLIS3*$^{-/-}$ cells at D30_L. Data are normalized to WT values (WT n=4, *GLIS3*$^{-/-}$ n=5). **(k)** qRT-PCR analysis of INS expression in FACS-purified WT and *GLIS3*$^{-/-}$ INS-GFP^{+} PP2-β cells (WT n=3, *GLIS3*$^{-/-}$ n=2). **(l)** Glucose-stimulated c-peptide secretion assay of WT and *GLIS3*$^{-/-}$ cells at D30_L. The data represents the fold change of c-peptide secretion in 20 mM (high) glucose condition vs. 2 mM (low) glucose condition. **(m)** C-peptide secretion in response to other β-cell secretagogues is shown for the *GLIS3*$^{-/-}$ cells at D30_L. The data represents the fold change of insulin secretion with 30 mM KCl or 30 μM Forskolin or 10 mM Arginine relative to KRBH treatment (n=5). P values by unpaired two-tailed t-test were *P<0.05, **P<0.01, ***P<0.001, ****P<0.0001. The center value is "mean". Error bar is SEM.

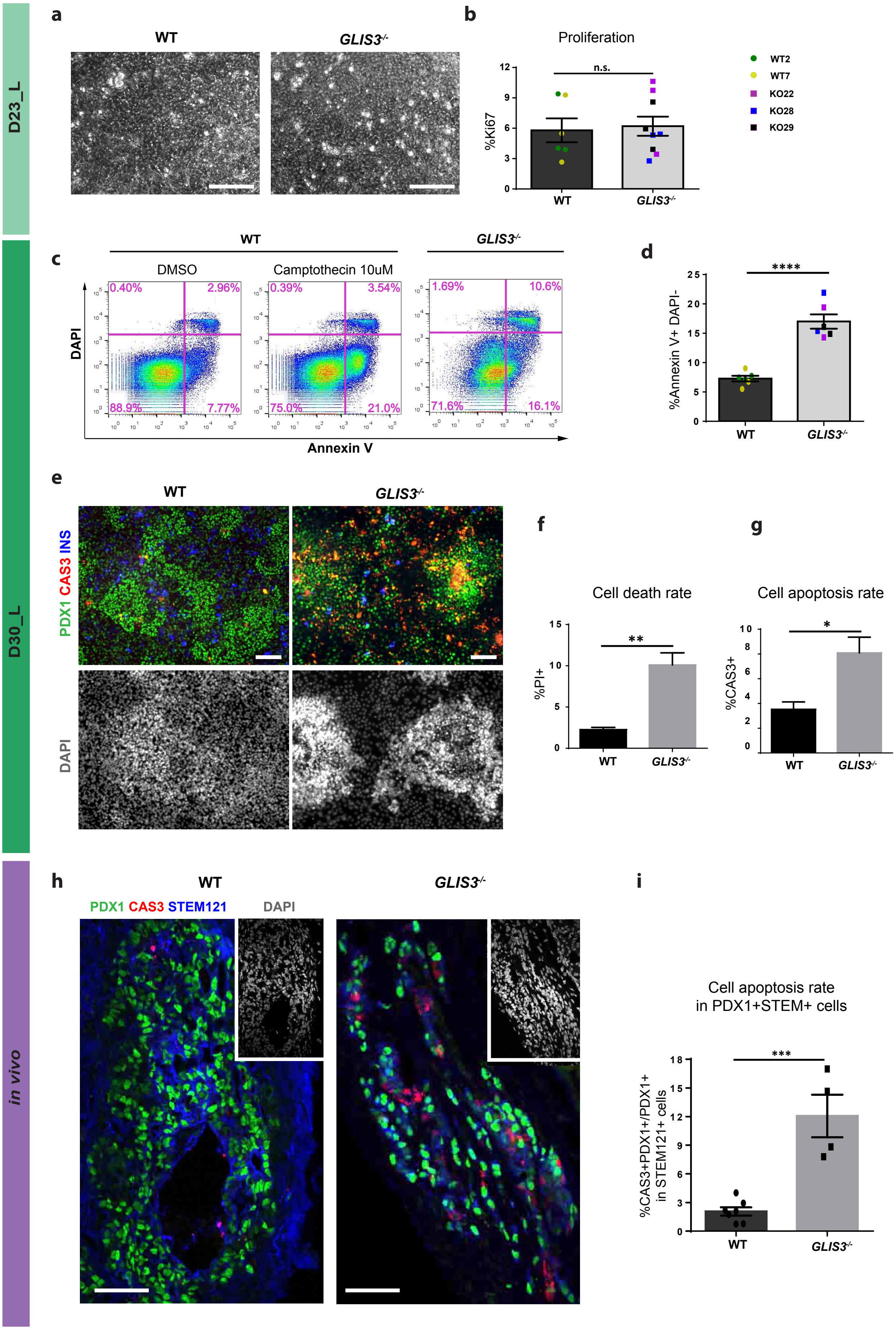

D23_L
D30_L
in vivo
a
WT
GLIS3-/-
b
Proliferation
n.s.
%Ki67
WT
GLIS3-/-
WT2
WT7
KO22
KO28
KO29
c
WT
GLIS3-/-
DMSO
Camptothecin 10uM
DAPI
Annexin V
0.40%
2.96%
88.9%
7.77%
0.39%
3.54%
75.0%
21.0%
1.69%
10.6%
71.6%
16.1%
d
****
%Annexin V+ DAPI-
WT
GLIS3-/-
e
WT
GLIS3-/-
PDX1 CAS3 INS
DAPI
f
Cell death rate
**
%PI+
WT
GLIS3-/-
g
Cell apoptosis rate
*
%CAS3+
WT
GLIS3-/-
h
WT
GLIS3-/-
PDX1 CAS3 STEM121
DAPI
i
Cell apoptosis rate
in PDX1+STEM+ cells
***
%CAS3+PDX1+/PDX1+
in STEM121+ cells
WT
GLIS3-/-

Supplementary Figure 5. Quantification of cell death in INS^- cells at D23_L and D30_L.

(a) Representative transmitted light images of WT and *GLIS3*$^{-/-}$ cells at D23_L (Scale bar= 100 µm). **(b)** Immunostaining quantification of $Ki67^+$ cells at D23_L (n=6). **(c, d)** Annexin V staining (c) and the quantification of early apoptotic rate (d, the percentage of Annexin V^+/$DAPI^-$ cells) at D30_L WT+DMSO, WT+10µM Camptothecin and *GLIS3*$^{-/-}$ cells (WT n=9, *GLIS3*$^{-/-}$ n=12). **(e)** Immunostaining for PDX1, cleaved caspase-3 and INS in WT and *GLIS3*$^{-/-}$ cells at D30_L. Scale bar=100 µm. **(f, g)** Quantification of cell death rate (f, the percentage of PI^+INS^-cells in INS^- cells) and apoptosis rate (g, the percentage of cleaved caspase-3^+INS^- cells in INS^- cells) in WT and *GLIS3*$^{-/-}$ cells at D30_L (n=3). **(h)** Immunohistochemistry for PDX1, cleaved caspase-3 and STEM121 in WT and *GLIS3*$^{-/-}$ cells transplanted *in vivo*. Scale bar=50 µm. **(i)** quantification of the percentage of apoptotic $PDX1^+$ cells ($CAS3^+PDX1^+STEM121^+$) in the $PDX1^+$ population within the graft ($PDX1^+STEM121^+$, WT n=7, *GLIS3*$^{-/-}$ n=4). *P* values by unpaired two-tailed t-test were $*P<0.05$, $**P<0.01$, $***P<0.001$, $****P<0.0001$. The center value is "mean". Error bar is SEM.

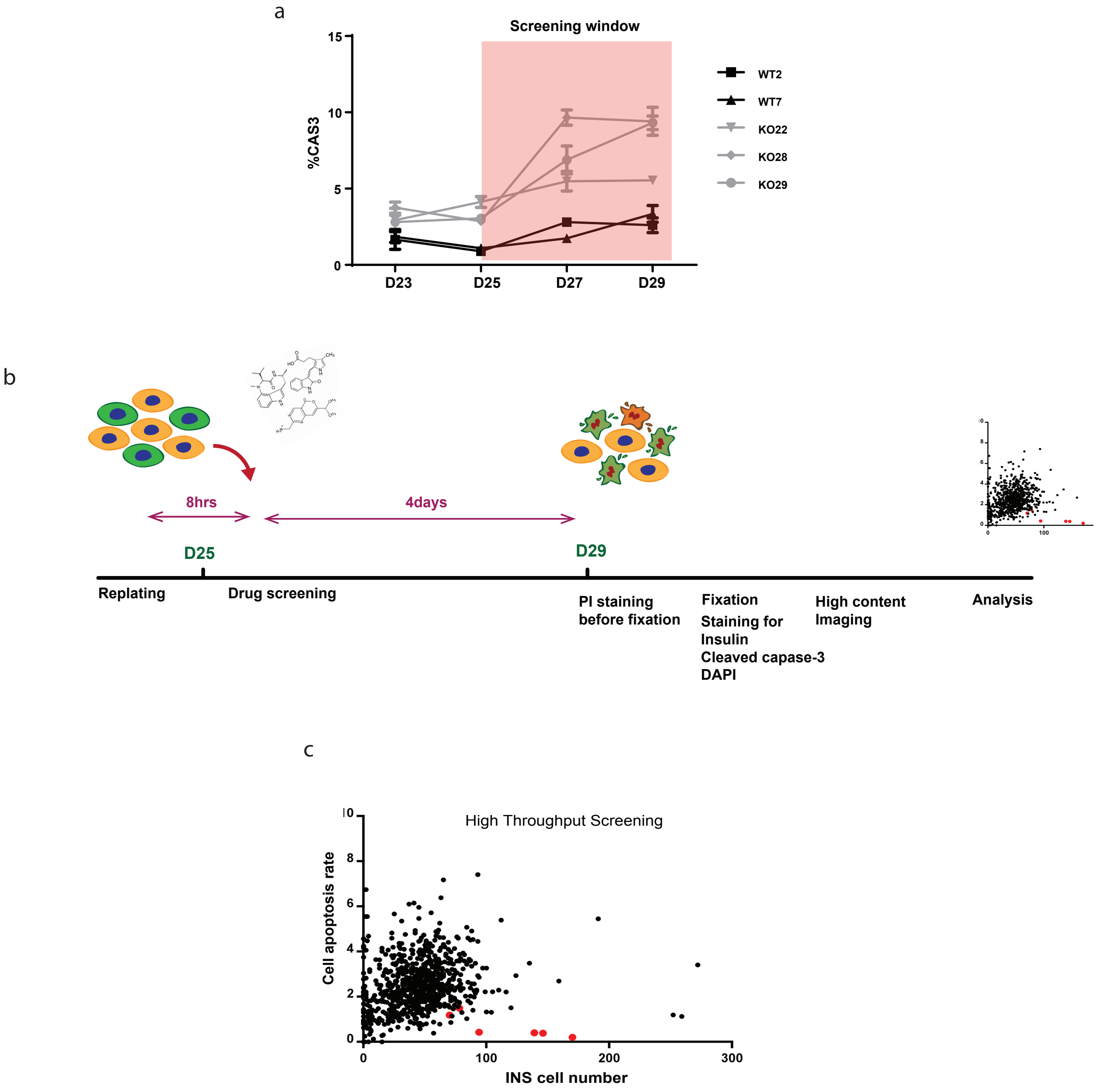

a
Screening window
%CAS3
15
10
5
0
D23
D25
D27
D29
WT2
WT7
KO22
KO28
KO29
b
8hrs
4days
D25
D29
Replating
Drug screening
PI staining before fixation
Fixation
Staining for Insulin
Cleaved capase-3
DAPI
High content Imaging
Analysis
c
High Throughput Screening
Cell apoptosis rate
10
8
6
4
2
0
0
100
200
300
INS cell number

Supplementary Figure 6. Design and primary results of the high throughput drug screen.

(a) Time-course staining to monitor the percentage of cleaved caspase-3^{+} cells in WT and *GLIS3*$^{-/-}$ cells to optimize the window for drug screening (n=2). **(b)** Schematic representation of the screening process. **(c)** Representative dot plot of the primary screening results. Red dots show confirmed hits.

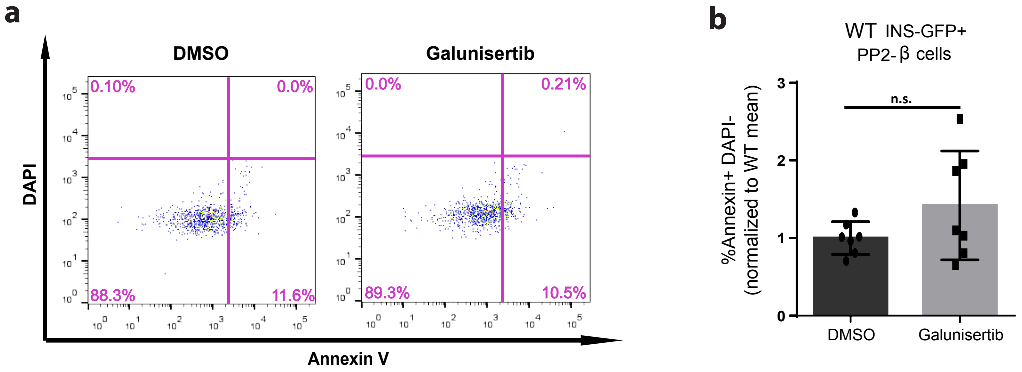
a
DMSO
Galunisertib
DAPI
Annexin V
0.10%
0.0%
88.3%
11.6%
0.0%
0.21%
89.3%
10.5%
b
WT INS-GFP+
PP2- β cells
%Annexin+ DAPI-
(normalized to WT mean)
n.s.
DMSO
Galunisertib

Supplementary Figure 7. Galunisertib does not affect cell apoptosis of WT PP2-β cells.

(a,b) Flow cytometry analysis (a) and quantification of the percentage (b) of Annexin V^+ cells in WT INS-GFP^+ PP2-β treated with DMSO or 10 μM galunisertib (n=7). n.s. not significant. The center value is "mean". Error bar is SEM.

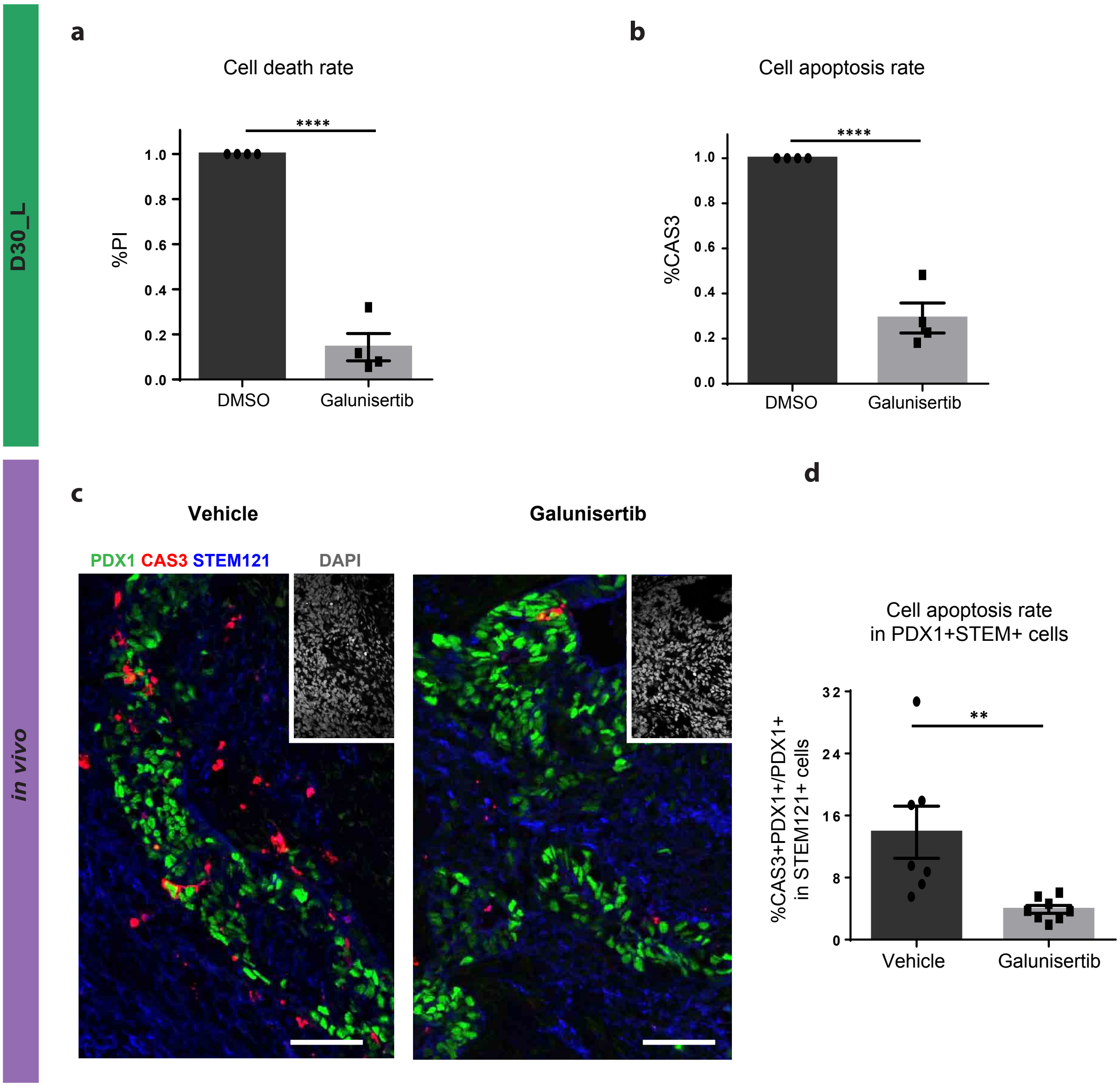
D30_L
a
Cell death rate
****
%PI
1.0
0.8
0.6
0.4
0.2
0.0
DMSO
Galunisertib
b
Cell apoptosis rate
****
%CAS3
1.0
0.8
0.6
0.4
0.2
0.0
DMSO
Galunisertib
in vivo
c
Vehicle
Galunisertib
PDX1 CAS3 STEM121
DAPI
d
Cell apoptosis rate
in PDX1+STEM+ cells
%CAS3+PDX1+/PDX1+
in STEM121+ cells
**
32
24
16
8
0
Vehicle
Galunisertib

Supplementary Figure 8. Effect of galunisertib and other TGFβ inhibitors on INS$^-$ *GLIS3*$^{-/-}$ at D30_L.

(a, b) Quantification of the cell death rate (a, the percentage of PI$^+$ cells) and apoptosis rate (b, the percentage of cleaved caspase-3$^+$ cells) of *GLIS3*$^{-/-}$ cells at D30_L treated with galunisertib or DMSO (n=4). **(c)** Immunohistochemistry for PDX1, cleaved caspase-3 and STEM121 in *GLIS3*$^{-/-}$ grafts treated with vehicle or galunisertib. Scale bar= 50 µm. **(d)** Quantification of the percentage of apoptotic PDX1$^+$ cells (CAS3$^+$PDX1$^+$STEM121$^+$) in the PDX1$^+$ population within the graft (PDX1$^+$STEM121$^+$, vehicle n=7, galunisertib n=8). *P* values by unpaired two-tailed t-test were $^{**}P<0.01$, $^{***}P<0.001$, $^{****}P<0.0001$. The center value is "mean". Error bar is SEM.

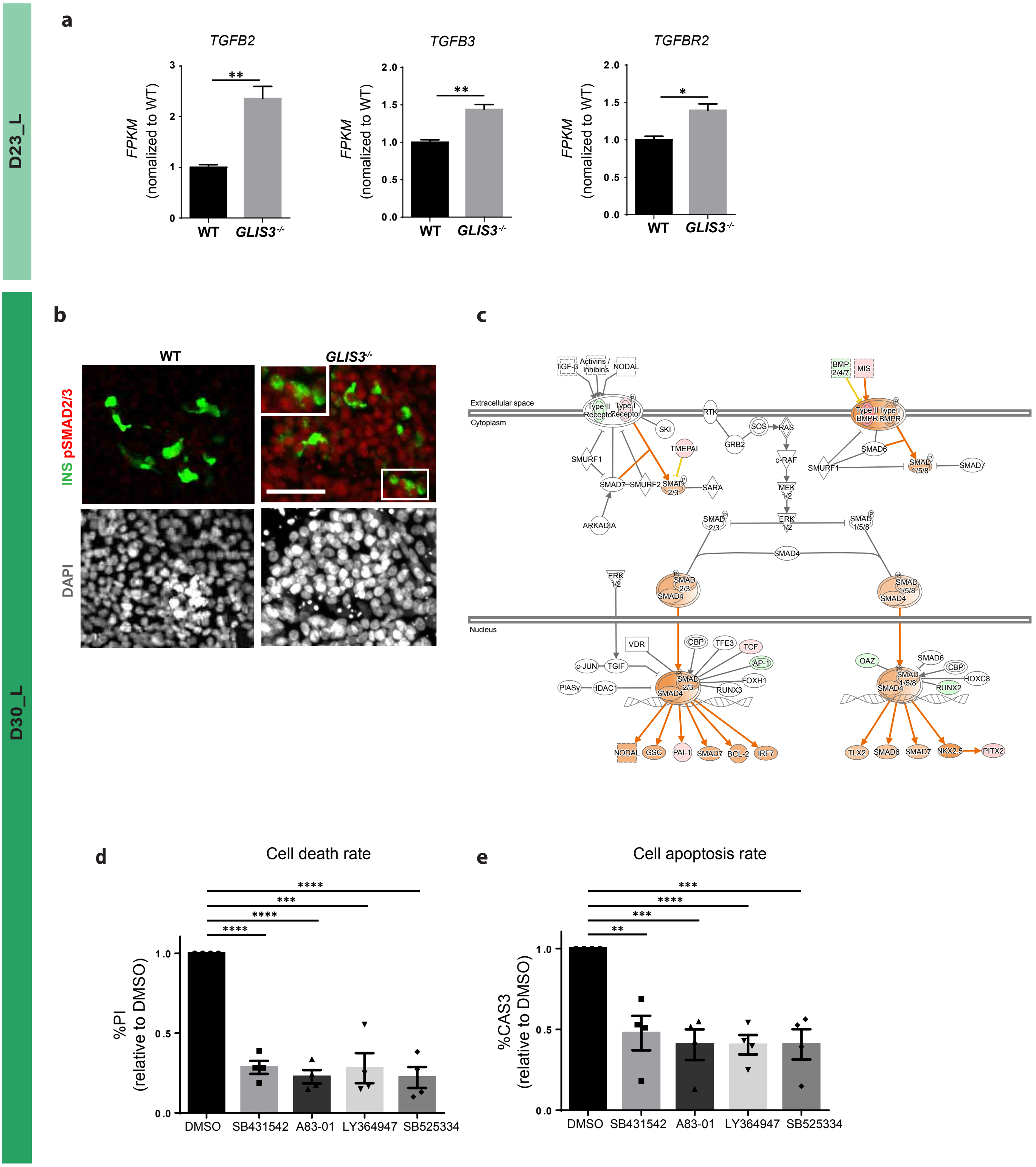
D23_L
a
TGFB2
TGFB3
TGFBR2
FPKM
(nomalized to WT)
WT
GLIS3-/-
**
*
D30_L
b
WT
GLIS3-/-
INS pSMAD2/3
DAPI
c
Extracellular space
Cytoplasm
Nucleus
d
Cell death rate
%PI
(relative to DMSO)
DMSO
SB431542
A83-01
LY364947
SB525334
****
***
e
Cell apoptosis rate
%CAS3
(relative to DMSO)
**

Supplementary Figure 9. ***GLIS3$^{-/-}$*** **pancreatic cells display increased TGFβ signaling.**

(a) FPKM values for TGFβ genes *TGFB2*, *TGFB3* and *TGFBR2* in WT and *GLIS3$^{-/-}$* PP2 cells (n= 3). **(b)** Immunostaining for pSMAD/3 and insulin WT and *GLIS3$^{-/-}$* PP2-β cells. Scale bar=50 µm.. **(c)** Ingenuity pathway analysis prediction of TGFβ signaling pathway activity in the WT and *GLIS3$^{-/-}$* INS-GFP^{+} PP2-β cells. **(d, e)** Quantification of the cell death rate (d, the percentage of PI^{+} cells) and cell apoptosis rate (e, the percentage of cleaved caspase-3^{+} cells) of *GLIS3$^{-/-}$* cells at D30_L treated with the indicated TGFβ inhibitors (n=4). *P* values by unpaired two-tailed t-test were $^{**}P<0.01$, $^{***}P<0.001$, $^{****}P<0.0001$. The center value is "mean". Error bar is SEM.

a

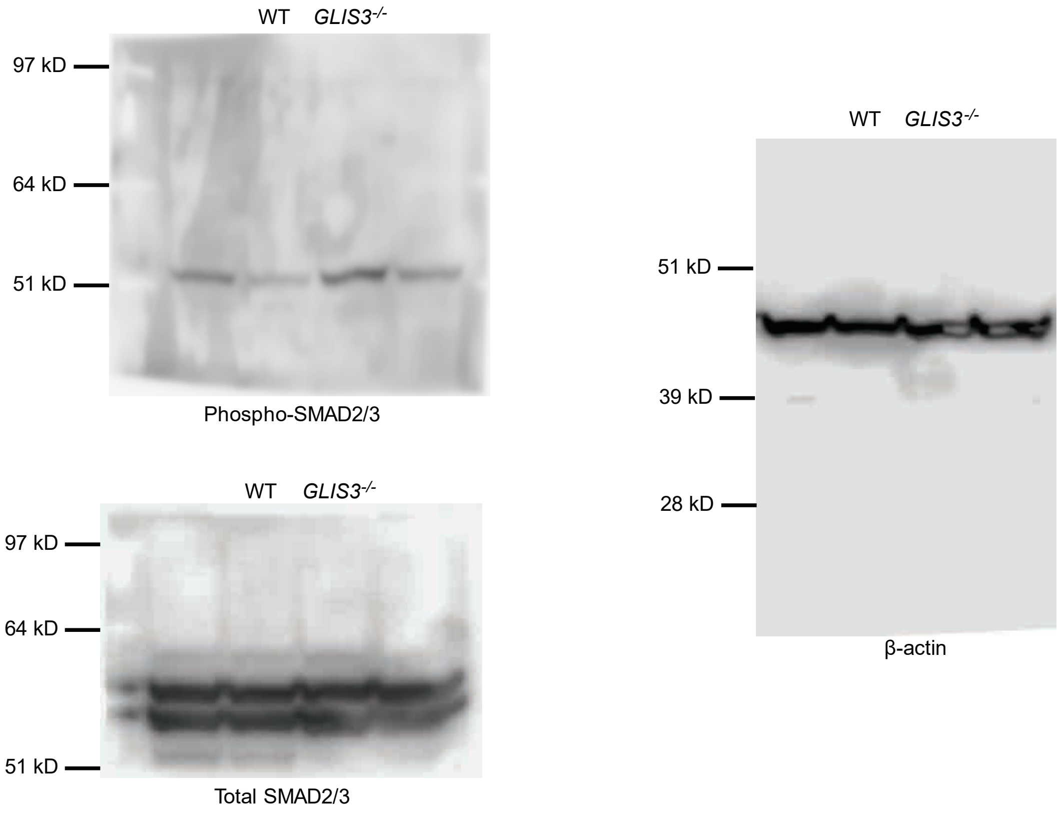
WT
GLIS3-/-
97 kD
64 kD
51 kD
Phospho-SMAD2/3
WT
GLIS3-/-
97 kD
64 kD
51 kD
Total SMAD2/3
WT
GLIS3-/-
51 kD
39 kD
28 kD
β-actin

b

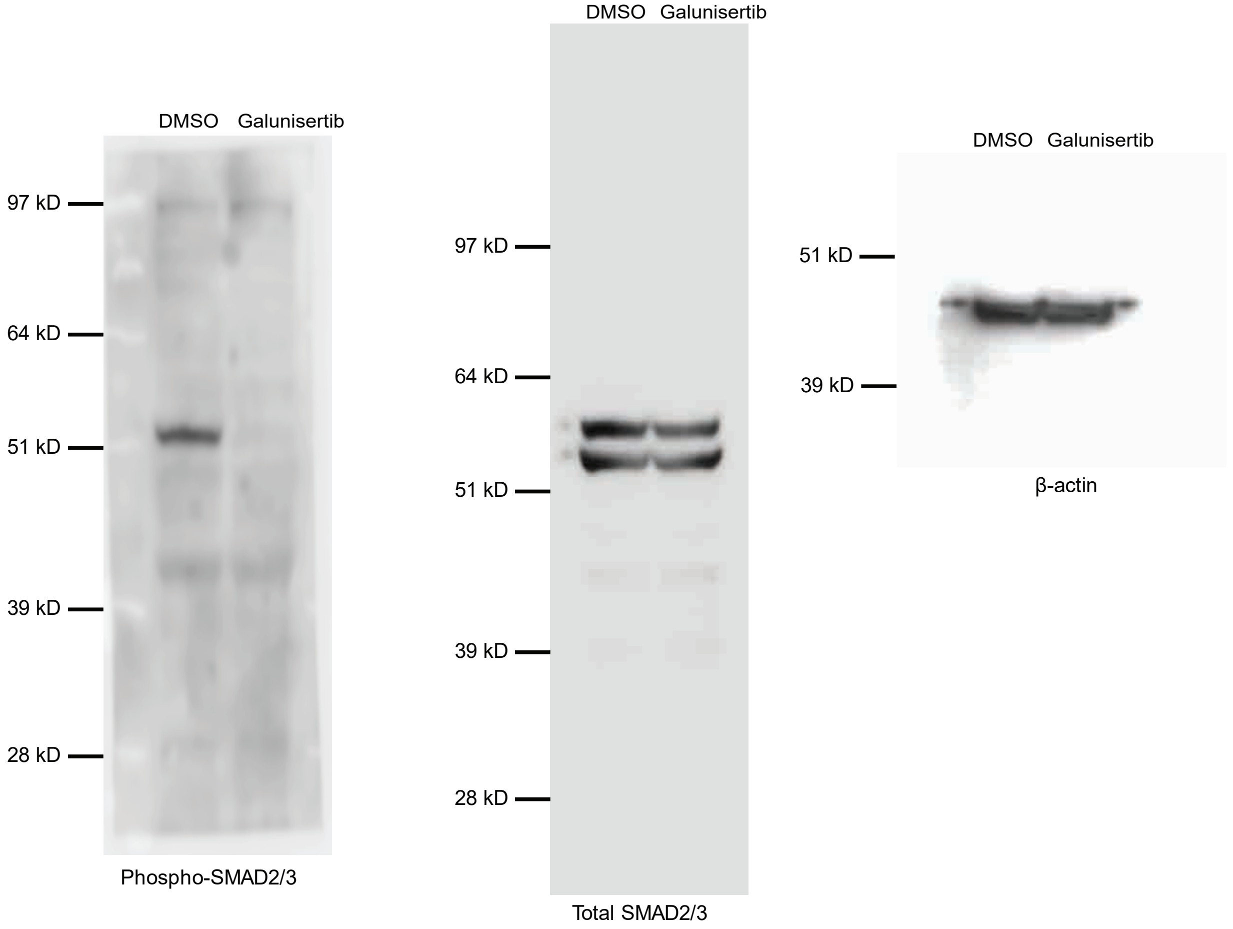
DMSO
Galunisertib
97 kD
64 kD
51 kD
39 kD
28 kD
Phospho-SMAD2/3
DMSO
Galunisertib
97 kD
64 kD
51 kD
39 kD
28 kD
Total SMAD2/3
DMSO
Galunisertib
51 kD
39 kD
β-actin

Supplementary Figure 10. Uncropped western blots of Figure 5.

(a) Related to Fig. 5c. **(b)** Related to Fig 5f.

Supplementary Table 1. Definition of differentiation stage and cells.

Stage	Cells
D9/day 9	PP1 cells
D16_E/day 16 using the early progenitor protocol	PP2 cells
D23_L/day 23 using the late progenitor protocol	INS-GFP^{+} cells were defined as PP1-β cells
D30_L/day 23 using the late progenitor protocol	INS-GFP^{+} cells were defined as PP2-β cells

Supplementary Table 2. QRT-PCR primers.

**Gene**	**Primer Sequence (5'-3')**	
ACTB	**F**	CAATGTGGCCGAGGACTTTG
	**R**	CATTCTCCTTAGAGAGAAGTGG
PDX1	**F**	CCTTTCCCATGGATGAAGTC
	**R**	CGTCCGCTTGTTCTCCTC
NKX6.1	**F**	TCGTTTGGCCTATTCGTTGG
	**R**	TGTCTCCGAGTCCTGCTTC
NEUROD1	**F**	ATGACCAAATCGTACAGCGAG
	**R**	GTTCATGGCTTCGAGGTCGT
MAFA	**F**	CTTCAGCAAGGAGGAGGTCATC
	**R**	CTCGTATTTCTCCTTGTACAGGTCC
GLIS3	**F**	GACTCACTCGGGCATTACAG
	**R**	TCCACGGTGCTGATCTGCAAG
UCN3	**F**	CCCACAAGTTCTACAAAGCCA
	**R**	TCCCGAAGAGGCGTCTCTG

Supplementary Table 3. hPSCs used in the study.

**hPSC line**	**Source**
$INS^{w/GFP}$ HES3	Stanley lab at Monash University
HUES8	Harvard University
H1	WiCell Research Institute

Supplementary Table 4. CRISPR sgRNA sequences.

Gene	sgRNA Sequence (5'-3')	
GLIS3-CRISPR-1	F	CACCGTCCCATGATGGTTCAGCGAC
	R	AAACGTCGCTGAACCATCATGGGAC
GLIS3-CRISPR-2	F	CACCGAGATCAGTCCTAGCTTACAG
	R	AAACCTGTAAGCTAGGACTGATCTC

Supplementary Table 5. PCR and sequencing primers used for genotyping the knockout hESC lines.

Gene	Primer Sequence (5'-3')	
GLIS3-seq	F	GGGTCCTGATATAAGCGTGC
	R	TCACTCACACCACAAGACAGT

Supplementary Table 6. Efficiency for the creation of biallelic knockout hESC lines.

Gene	Sequenced Subclones	Monoallelic knockout clones	Biallelic knockout clones	Biallelic knockout efficiency
GLIS3	30	2	24	80%

Supplementary Table 7. Clonal lines used for each experiment.

Figure	WT lines used	KO lines used
2A	WT(PL)*, WT2	KO28, KO29
2A-MAFA	WT2, WT7, WT(PL)	KO22, KO28, KO29
2B	WT(PL), WT2	KO28, KO29
2C	WT(PL)	KO29
2D, 2E	WT(PL), WT2	KO28, KO29
2F, 2G	WT2, WT7	KO22, KO28, KO29
2H-2J	WT2, WT7, WT(PL)	KO22, KO28, KO29
2K	WT2	KO22
2L, 2M	WT2, WT7, WT(PL)	KO22, KO28, KO29
2N	WT2, WT7, WT(PL)	KO22, KO29
3A	WT2, WT7, WT(PL)	KO22, KO28, KO29
3B	WT2	KO29
3C	WT7	KO29
3D	WT2	KO22
3E	WT2, WT7	KO22, KO28, KO29
3F	WT2, WT7	KO22, KO28, KO29
3G	WT(PL)	KO29
3H	WT2, WT7, WT(PL)	KO22, KO28, KO29
3I-3L	WT2	KO29
4C	-	KO29
4D	-	KO22
4E, 4F	-	KO22, KO28, KO29

4G	-	
4H, 4I	-	KO22, KO28, KO29
4J-4M	-	KO29
5A, 5B	WT(PL), WT2	KO28, KO29
5C, 5D	WT2	KO22
5E	WT2, WT(PL)	KO28, KO29
5F, 5G	-	KO22
5H, 5I	-	KO22, KO28, KO29
S2A-S2L	WT2, WT7, WT(PL)	KO22, KO28, KO29
S3A	WT(PL), WT2	KO28, KO29
S3B-S3F	WT2, WT7	KO22, KO28, KO29
S3G, S3H	WT2	KO29
S3I	WT2, WT7	KO22, KO28, KO29
S4A, S4C, S4E	WT2, WT7	KO22, KO28, KO29
S4B, S4D, S4F	WT2, WT7, WT(PL)	KO22, KO28, KO29
S4G, S4H	WT2, WT7	KO22, KO28, KO29
S4I, S4J	WT2, WT7	KO22, KO28, KO29
S4K	WT2	KO29
S4L, S4M	WT2, WT7, WT(PL)	KO22, KO28, KO29
S5A	WT2	KO22
S5B	WT2, WT7	KO22, KO28, KO29
S5C	WT2	KO29
S5D	WT2, WT7	KO22, KO28, KO29
S5E	WT2	KO22

S5F, S5G	WT2, WT7	KO22, KO28, KO29
S5H, S5I	WT2	KO29
S6A	WT2, WT7	KO22, KO28, KO29
S6C	WT2 (control wells)	KO29
S7A	WT2	-
S7B	WT2, WT7, WT(PL)	-
S8A, S8B	-	KO22, KO28, KO29
S8C, S8D	-	KO29
S8E, S8F	-	KO22, KO28, KO29
S9A	WT2, WT(PL)	KO28, KO29
S9B	WT2	KO29
S9C	WT2, WT(PL)	KO28, KO29

Supplementary Table 8. Top hit compounds from the drug screening. Z-scores were calculated for the cleaved caspase-3 percentage using the formula Z=(x-μ)/σ.

Compound name	Z-score	Bioactivity
SB-525334	-2.33797	TGF-β RI Kinase Inhibitor VIII
LY2157299 (Galunisertib)	-2.2971	TGF-β RI Kinase Inhibitor
Protriptyline HCl	-2.26557	Norepinephrine uptake blocker
Suxibuzone	-2.15349	Analgesic, Anti-inflammatory
A83-01	-2.14045	TGF-beta RI Inhibitor IV
(±)-Ibuprofen	-2.13479	COX inhibitor, Anti-inflammatory
Hesperetin	-2.12035	P450 inhibitor
PD169316	-1.97457	P38 MAPK inhibitor
Betamethasone Valerate	-1.88811	Glucocorticoid
Ambroxol HCl	-1.85367	Expectorant
LY 364947	-1.59577	ALK5 Inhibitor I TGF-β RI Kinase Inhibitor

Supplementary Table 9. Summary of TGFβ inhibitors used in Figure 5.

Compound	Activity
SB 431542	Potent and selective inhibitor of the transforming growth factor-β (TGF-β) type I receptor activin receptor-like kinase ALK5 and its relatives ALK4 and ALK7
A83-01	Potent inhibitor of TGF-β type I receptor ALK5 kinase, type I activin/nodal receptor ALK4 and type I nodal receptor ALK7.<br>Weak inhibitor of ALK-1, -2, -3, -6 and MAPK activity
SB 525334	Selective inhibitor of transforming growth factor-β receptor I (ALK5, TGF-βRI)
LY-364947	Selective inhibitor of TGF-β type-I receptor (TGF-β RI, TGFR-I, TβR-I, ALK-5)
